# The Sigma Factor AlgU Regulates Exopolysaccharide Production and Nitrogen-Fixing Biofilm Formation by Directly Activating the Transcription of *pslA* in *Pseudomonas stutzeri* A1501

**DOI:** 10.3390/genes13050867

**Published:** 2022-05-12

**Authors:** Yahui Shao, Changyan Yin, Fanyang Lv, Shanshan Jiang, Shaoyu Wu, Yueyue Han, Wei Xue, Yiyuan Ma, Juan Zheng, Yuhua Zhan, Xiubin Ke, Wei Lu, Min Lin, Liguo Shang, Yongliang Yan

**Affiliations:** 1 Biotechnology Research Institute, Chinese Academy of Agricultural Sciences, Beijing 100081, China; 82101181002@caas.cn (Y.S.); 82101212015@caas.cn (C.Y.); lvfanyang@caas.cn (F.L.); 82101191004@caas.cn (S.J.); 82101192010@caas.cn (S.W.); 82101192070@caas.cn (Y.H.); 82101202011@caas.cn (W.X.); 82101201004@caas.cn (Y.M.); 82101202012@caas.cn (J.Z.); zhanyuhua@caas.cn (Y.Z.); kexiubin@caas.cn (X.K.); luwei01@caas.cn (W.L.); linmin@caas.cn (M.L.); 2 School of Basic Medicine, GuangXi University of Chinese Medicine, Nanning 530200, China

**Keywords:** nitrogen fixation, biofilm formation, the *algU* gene, the *pslA* gene, *Pseudomonas stutzeri*

## Abstract

*Pseudomonas stutzeri* A1501, a plant-associated diazotrophic bacterium, prefers to conform to a nitrogen-fixing biofilm state under nitrogen-deficient conditions. The extracytoplasmic function (ECF) sigma factor AlgU is reported to play key roles in exopolysaccharide (EPS) production and biofilm formation in the *Pseudomonas* genus; however, the function of AlgU in *P. stutzeri* A1501 is still unclear. In this work, we mainly investigated the role of *algU* in EPS production, biofilm formation and nitrogenase activity in A1501. The *algU* mutant Δ*algU* showed a dramatic decrease both in the EPS production and the biofilm formation capabilities. In addition, the biofilm-based nitrogenase activity was reduced by 81.4% in the Δ*algU* mutant. The transcriptional level of *pslA*, a key Psl-like (a major EPS in A1501) synthesis-related gene, was almost completely inhibited in the *algU* mutant and was upregulated by 2.8-fold in the *algU*-overexpressing strain. A predicted AlgU-binding site was identified in the promoter region of *pslA*. The DNase I footprinting assays indicated that AlgU could directly bind to the *pslA* promoter, and β-galactosidase activity analysis further revealed mutations of the AlgU-binding boxes drastically reduced the transcriptional activity of the *pslA* promoter; moreover, we also demonstrated that AlgU was positively regulated by RpoN at the transcriptional level and negatively regulated by the RNA-binding protein RsmA at the posttranscriptional level. Taken together, these data suggest that AlgU promotes EPS production and nitrogen-fixing biofilm formation by directly activating the transcription of *pslA*, and the expression of AlgU is controlled by RpoN and RsmA at different regulatory levels.

## 1. Introduction

When bacteria adhere to solid surfaces, they produce many extracellular polymeric substances in the extracellular matrix in response to various environmental signals, such as stress signals, and then the bacteria are embedded in a complicated three-dimensional structure called a biofilm [1,2]. It is well accepted that compared to the planktonic state, biofilms can help bacteria adapt to stressful environments. The high cell density in biofilms allows bacteria to adjust their gene expression and phenotypes via the quorum sensing system [3]. Exopolysaccharides are the major component of the biofilm matrix [4]; their composition presumably varies from organism to organism, and it is a key factor influencing biofilm biomass, structure and functions [4]. 

*Pseudomonas aeruginosa* is a model organism for studies of biofilm formation and exopolysaccharide production [5,6]. At least three exopolysaccharides (alginate, psl, and pel) are produced by *P. aeruginosa*. *P. aeruginosa* strains isolated from chronic infection patients with cystic fibrosis (CF) usually overproduce alginate and are called mucoid strains. In non-mucoid strains, alginate production is repressed, and psl and pel are the primary exopolysaccharides [7]. Alginate production is regulated by complicated regulatory and biosynthetic operons. The major regulatory operon (*algU-mucABCD*) encodes the sigma factor AlgU and the anti-sigma factor MucA [8,9]. AlgU activates the transcription of *algD* and other alginate biosynthetic genes by directly binding the AlgU-binding sites (GAACTT-N16/17-TCCAA) in the promoters [10]. It is also reported that AlgU plays a key role in psl production in *P. aeruginosa* PAO1; however, promoter mapping indicated that AlgU is probably not directly responsible for the transcription of the *psl* operon [11]. In addition to the regulation of alginate and psl biosynthesis, AlgU in *P. aeruginosa* also contributes to environmental stress tolerance and virulence [12,13]. In other *Pseudomonas* species, the functions of AlgU are also characterized. It was reported that AlgU regulates the motility and translocator expression in *P. fluorescens* and controls the abiotic stress tolerance and virulence in *P. syringae* [14,15,16].

*P. stutzeri* A1501 is a plant-associated bacterium that colonizes the root surface and endophytically invades the root tissues of host plants [17,18]. A1501 fixes nitrogen and promotes plant growth [19,20]. Analysis of the complete genome sequence enables easy identification of the key genes for nitrogen fixation and other important phenotypes, such as biofilm formation. The functions of the AmtB–GlnK–NtrBC-RpoN regulatory cascade have been well studied in detail, and several regulatory ncRNAs that control the expression of *nif* genes in response to nutrient stress have been reported [21,22,23,24].

Recently, biofilm formation was linked to nitrogen fixation in A1501. Wang et al. reported the effect of physiological conditions on biofilm formation and the architecture of nitrogen-fixing biofilms. In addition, biofilms could enable bacteria to fix nitrogen under aerobic conditions [25]. Comparative genomics analysis showed that A1501 does not possess the complete alginate and pel systems, but it contains genes possibly involved in a psl-like biosynthesis operon [18]. Shang et al. investigated the Rpo-Gac-Rsm regulatory network for nitrogen-fixing biofilm formation in A1501. Notably, *pslA* plays an essential role in the production of Psl-like exopolysaccharides, and RpoN directly activates the transcription of *pslA* [26]. An *algU* homologous gene, encoded by PST1223, was identified in A1501, however, its function is still unclear. In the present study, an *algU* mutant strain and an *algU* overexpression strain were constructed and an AlgU dependent regulation network on biofilm formation was established. The phenotype analysis indicated that AlgU played important roles in exopolysaccharide production, biofilm formation and nitrogen fixation. Results from qRT-PCR assays and β-galactosidase activity assays, together with DNase I footprinting assays confirmed that AlgU directly activated the transcription of *pslA* and AlgU was positively regulated by RpoN at the transcriptional level and negatively regulated by the RNA-binding protein RsmA at the posttranscriptional level.

## 2. Materials and Methods

### 2.1. Bacterial Strains and Medium

The bacterial strains and plasmids used in this study are shown in Table S1. *P. stutzeri* wild-type A1501 and its derivatives were grown in LB medium or minimal K medium (KH_2_PO_4_ 0.4 g·L^−1^, K_2_HPO_4_ 0.1 g·L^−1^, NaCl 0.1 g·L^−1^, MgSO_4_ 0.01 g·L^−1^, MnSO_4_ 0.01 g·L^−1^, Fe_2_(SO_4_)_3_·H_2_O 0.01 g·L^−1^, Na_2_MoO_4_·H_2_O 0.01 g·L^−1^ pH 6.8) supplemented by the desired carbon and nitrogen sources indicated in the text at 30 °C in shake flasks at 220 rpm. *Escherichia coli* strains were grown at 37 °C in LB medium. Antibiotics were used at the following concentrations: kanamycin (Km): 50 μg·mL^−1^, tetracycline (Tc): 10 μg·mL^−1^, and chloromycetin (Cm): 34 μg·mL^−1^.

### 2.2. Construction of the algU Mutant, Complemented Strain and Overexpression Strain

The *algU gene* in *P. stutzeri* A1501 was inactivated with the suicide plasmid vector pK18mob [27]. The primers were designed to amplify the internal 200 bp fragment of the *algU* gene by PCR (Table S2). The PCR product was cloned into pK18mob with a recombinase (Vazyme Biotech Co., Ltd., Nanjing, China), and the product was named pK18mob-*algU*. pK18mob-*algU* was introduced into A1501 by triparental mating with the help of the pRK2013 vector [28], generating an *algU* nonpolar insertion mutant strain. The correct *algU* mutant strain was confirmed by PCR testing and DNA sequencing. A DNA fragment containing the *algU* gene region with its native promoter (500 bp) was cloned into pLAFR3, a derivative of the low-copy-number wide-host-range plasmid RK2 [29]. The constructed plasmid was named pL*algU* and individually introduced into the *algU* mutant and A1501 via triparental mating, generating the *algU-*complemented strain and *algU* overexpression strain.

### 2.3. Exopolysaccharide Isolation and Concentration Measurement

*P. stutzeri* wild-type A1501 and its derivatives were grown (30 °C, 220 rpm) in a minimal K medium containing 50 mM lactate and 6 mM NH_4_Cl. The cultures were centrifuged to collect the supernatants, and then exopolysaccharides were precipitated and isolated from the supernatants by adding two volumes of cold absolute ethanol. The purified exopolysaccharide was dissolved by ddH_2_O, and the exopolysaccharide content of samples was measured quantitatively by using the phenol-sulfuric acid method [30]. Final concentrations were expressed as milligrams of exopolysaccharide per gram of bacterial protein.

### 2.4. Biofilm Formation Assay

A biofilm formation assay using the crystal violet (CV) method was performed according to a previous method [26]. *P. stutzeri* wild-type A1501 and selected strains were grown (30 °C, 220 rpm) overnight in LB medium. The cells were collected by centrifugation and resuspended in a nitrogen-free minimal K medium containing 50 mM lactate. The OD_600_ of the cell suspensions was adjusted to 0.2 with a nitrogen-free K medium containing 50 mM lactate. A total of 160 μL of each suspension was dropped into the wells in a 96-well PVC microtiter plate. The microtiter plate wrapped with parafilm was placed without agitation in an incubator for 48 h. At the end of incubation, the non-adhered planktonic cells were gently removed using a pipette, and the wells were washed twice with ddH_2_O. Then, 0.1% CV solution in ethanol was used to stain the biofilm in the well. The free CV in the wells was washed away with ddH_2_O, and the well-adhered CV was solubilized with 30% acetic acid and quantified by measuring the OD_560_ of the resulting solution using a microplate reader.

### 2.5. Nitrogenase Activity Assay

Biofilm-based nitrogenase activity was measured with the acetylene reduction test according to a previous method, with modifications [31]. *P. stutzeri* strains were grown overnight in LB medium, and then, cells were collected and washed twice with nitrogen-free K medium containing 50 mM lactate. The OD_600_ of the cell suspension was diluted to 0.2 with nitrogen-free K medium containing 50 mM lactate, and 10 mL of the cell suspension was removed into a 110 mL flask under aerobic conditions (21% oxygen) and was incubated for 48 h at 30 °C under static conditions and then 10% acetylene was added. The gas samples (0.25 mL) were taken from the flask at regular intervals (4, 6, 8 and 10 h) and were analyzed on a poly divinylbenzene porous bead GDX-502 column using a gas chromatograph SP-2100 with a flame ionization detector (Beijing Beifen-Ruili Analytical instrument Co., Ltd., Beijing, China). The ethylene content in the gas sample was calculated by reference to an ethylene standard. The nitrogenase activity was expressed as nanomoles of ethylene (mg protein × h)^−1^. The concentration of total protein was measured using the Bradford protein assay kit (Bio-Rad Laboratories Inc., Hercules, CA, USA).

### 2.6. RNA Isolation and qRT-PCR Assay

To analyze the gene expression in wild-type A1501, mutant strain Δ*algU* or *algU*-overexpressing strain A1501(pL*algU*), bacteria were grown under tested conditions as described. Cells were harvested and centrifuged for 10 min at 4 °C, and pellets were quick-frozen in liquid nitrogen and stored at −80 °C. Total RNA was isolated with an innuPREP RNA Mini Kit (Analytik Jena, Berlin, Germany) following the manufacturer’s instructions. RNA reverse transcription was conducted using a reverse transcription kit (HiScript^®^ II 1st Strand cDNA Synthesis Kit (+gDNA wiper), Vazyme Biotech Co., Ltd., Nanjing, China). Genomic DNA contamination was quickly and completely removed by treatment with gDNA wiper Mix. The genomic DNA contamination was assessed by using primer pair *nifL*-*rnfA* F/R (Table S2). Aliquots of 1 μg of total RNA from each sample were reverse-transcribed to cDNA and the final product was diluted to 100 ng μL^−1^ in RNase-free water. The gene-specific primers listed in Table S2 for quantitative real-time (qRT) PCR were designed with the primer-blast tool on the NCBI website, and the 16S rRNA gene was used as the endogenous reference to normalize the expression of the target genes in different cDNAs. The universality of these primer pairs was tested by melt curve analysis. qRT-PCR was carried out with a ChamQ SYBR qPCR master mix kit (Vazyme Biotech Co., Ltd., Nanjing, China) using an ABI 7500 real-time PCR system according to the manufacturers’ recommendations. The relative expression levels of target genes were analyzed with the 2^–ΔΔCt^ method.

### 2.7. β-Galactosidase Activity Assay

To test the transcriptional expression of the native or mutant promoter of *pslA*, the 500 bp regions upstream of the transcription start site were cloned into the promoterless *lacZ* reporter plasmid pGD926 [32], and the resulting plasmids were introduced into *P. stutzeri* wild-type A1501 by triparental mating. The β-galactosidase activity assay was performed according to a previous method [33]. *P. stutzeri* strains carrying the *lacZ* fusion were grown (30 °C, 220 rpm) overnight in an LB medium. The cells were collected by centrifugation and washed twice with a nitrogen-free K medium containing 50 mM lactate. Then, the OD_600_ of the cell suspensions was adjusted to 0.1 with a nitrogen-free K medium containing 50 mM lactate. Ten millilitres of the cell suspension were removed into a 60 mL flask. The cell suspension was incubated for 4 h at 30 °C with shaking under a nitrogen fixation atmosphere containing 0.5% oxygen. After incubation, the cells were collected by centrifugation and resuspended in 1.0 mL of Z-buffer in 2.0 mL EP tubes. Two to three drops of chloroform were added into the tubes and mixed by inverting several times to lyse cells. The tubes were placed with tops open in an incubator at 30 °C for 40 min. Two hundred microliters of ONPG solution (4 mg/mL) were added to the tubes, and the time of colour change was recorded. The reaction was stopped with 500 μL of 1 M Na_2_CO_3_ solution. Then, 150 μL of the reaction solution was added to a 96-well microplate. The OD_420_ and OD_550_ were measured using a microplate reader. The β-Galactosidase activity was expressed as Miller units [33].

### 2.8. Western Blot Assays for algU-flag Expression

The *algU* gene region containing the 500 bp native promoter was cloned by PCR with the designed primers (*algU*-flag-F/R) (Table S2). The *algU*-flag-R primer was added with the flag tag sequence. The *algU*-flag PCR amplicons were cloned into the pGD926 vector with recombinase, and the product was named pG*algU*-flag. pG*algU*-flag was then introduced into *P. stutzeri* wild-type A1501 or selected mutants by triparental mating with the helper vector pRK2013. Western blotting was performed using protein extracts of bacterial cells grown for 10 h in K medium containing 50 mM lactate and 6 mM NH_4_Cl. Five micrograms of total protein were loaded and separated by SDS polyacrylamide gel electrophoresis (SDS-PAGE). Protein concentrations were determined using the Bio-Rad protein assay reagent kit. The separated protein bands were transferred to a polyvinylidene difluoride (PVDF) membrane (GE Healthcare, Piscataway, NJ, USA). The membrane was incubated with a monoclonal antibody against the flag peptide (Sigma-Aldrich Co. LLC, Saint Louis, MO, USA) for 12 h at 4 °C and then washed three times in TBS/Tween buffer before being incubated with an anti-mouse secondary antibody (Sangon Biotech Co., Ltd., Shanghai, China) for 2 h. Detection was performed using an HRP-DAB chemistry kit (Tiangen Biotech Co., Ltd., Beijing, China).

### 2.9. Determination of Transcriptional Start Site by 5′RACE

The rapid amplification of cDNA ends (5′ RACE) method (Invitrogen Corporation, Carlsbad, CA, USA) was used to determine the transcriptional start site for *algU.* First, first-strand cDNA was synthesized with the GSP1 primer (Table S2), which was specific for the *algU* sequence. The purified cDNA was tailed with dCTP by terminal deoxynucleotidyl transferase. PCR amplification was performed with the sequence-specific primer GSP2 and the anchor primer AAP (Table S2). The 5′ RACE PCR products were cloned into the pGEM-T Easy vector (Promega (Beijing) Biotech Co., Ltd., Beijing, China) and sequenced to map the 5′ end of the transcript.

### 2.10. DNase I Footprinting Assay

DNase I footprinting assay was performed by Tolo Biotech according to a method previously described [34]. For the preparation of fluorescent FAM labelled probes, the *pslA* promoter region was PCR amplified from the plasmid pJET1.2-*pslA* using primers of pJET1.2F2 and pJET1.2R2 (FAM). For each assay, 450 ng probes were incubated with different amounts of protein AlgU in a total volume of 40 µL. After incubation for 30 min at 30 °C, 10 µL solution containing about 0.015 unit DNase I (Promega (Beijing) Biotech Co., Ltd., Beijing, China) and 100 nmol freshly prepared CaCl_2_ was added and further incubation was performed at 37 °C for 1 min. The reaction was stopped by adding 140 µL DNase I stop solution (200 mM unbuffered sodium acetate, 30 mM EDTA and 0.15% SDS). Samples were firstly extracted with phenol/chloroform, then precipitated with ethanol. Pellets were dissolved in 30 µL MilliQ water. The preparation of the DNA ladder, electrophoresis and data analysis were the same as described before [34], except that the GeneScan-LIZ600 size standard (Applied Biosystems, Inc., Foster City, CA, USA) was used.

### 2.11. Purification of RpoN Protein and Gel Mobility-Shift Assay for RpoN Protein and algU Promoter DNA

The His-tagged RpoN was produced by the pET-28a expression system based on the previous method [26]. The DNA probes for gel mobility-shift assay were firstly prepared by PCR-amplifying (300-bp *algU* promoter region using the primers EMSA-*algU*-F/ EMSA-*algU*-R, 300-bp *nifA* promoter region using the primers EMSA- *nifA*-F/ EMSA- *nifA*-R, 300-bp negative control DNA using the primers EMSA-*nc*-F/ EMSA-*nc*-R) (Table S2) and was cloned into the clone vector pJET1.2. The labelled DNA probes were produced by PCR-amplifying using the FAM-labelled primers pJET1.2-F (FAM) / pJET1.2-R (Table S2). For each gel mobility-shift assay, 50 ng DNA probes were incubated with purified RpoN protein (0 μg, 2 μg, 5 μg, 10 μg) in a total volume of 20 μL (50 mM Tris-HCl [pH 8.0], 100 mM KCl, 2.5 mM MgCl_2_, 0.2 mM DTT, 2 μg poly (dI-dC) and 10% glycerol). After incubation for 30 min at 30 °C, the reaction system was loaded into 4% PAGE gel buffered with 0.5 × TBE. Gels were scanned with ImageQuant LAS 4000 mini (GE Healthcare Bio-Sciences AB, Uppsala, Sweden).

### 2.12. Purification of RsmA Protein and Gel Mobility-Shift Assay for RsmA Protein and algU 3′-UTR RNA

The RsmA protein was expressed and purified according to a previous method [26]. The 28 bp *algU* 3′-UTR ssRNA oligonucleotides were synthesized in GenePharma Co., Ltd., Shanghai, China. The binding reaction for purified RsmA protein and target RNA was carried out at room temperature in the binding buffer (10 mM HEPES pH 7.9, 35 mM KCl, 2 mM MgCl_2_); 10 μL binding reactant was loaded into the 5% non-denaturing polyacrylamide gel and the electrophoresis was performed at 4 °C. The RNA was removed from the gel to the nylon membrane with the SEM-DRY transfer cell (Bio-Rad Laboratories Inc., Hercules, CA, USA). Then, RNA was cross-linked into the nylon membrane and was hybridized with the specified ssDNA oligonucleotides labelled with digoxin. The RNA detection was carried out using the X-film with the DIG northern starter kit (Roche Diagnostics Corporation, Indianapolis, IN, USA) according to the manufacturer’s instructions.

### 2.13. Statistical Analysis

Statistical analysis was performed by one-way ANOVA. *p*-values of 0.05 or less were considered statistically significant. Error bars represent the standard deviation (SD) of the three biological replicates from a single experiment.

## 3. Results

### 3.1. algU Positively Regulated the Exopolysaccharide Production

**Figure 1:**
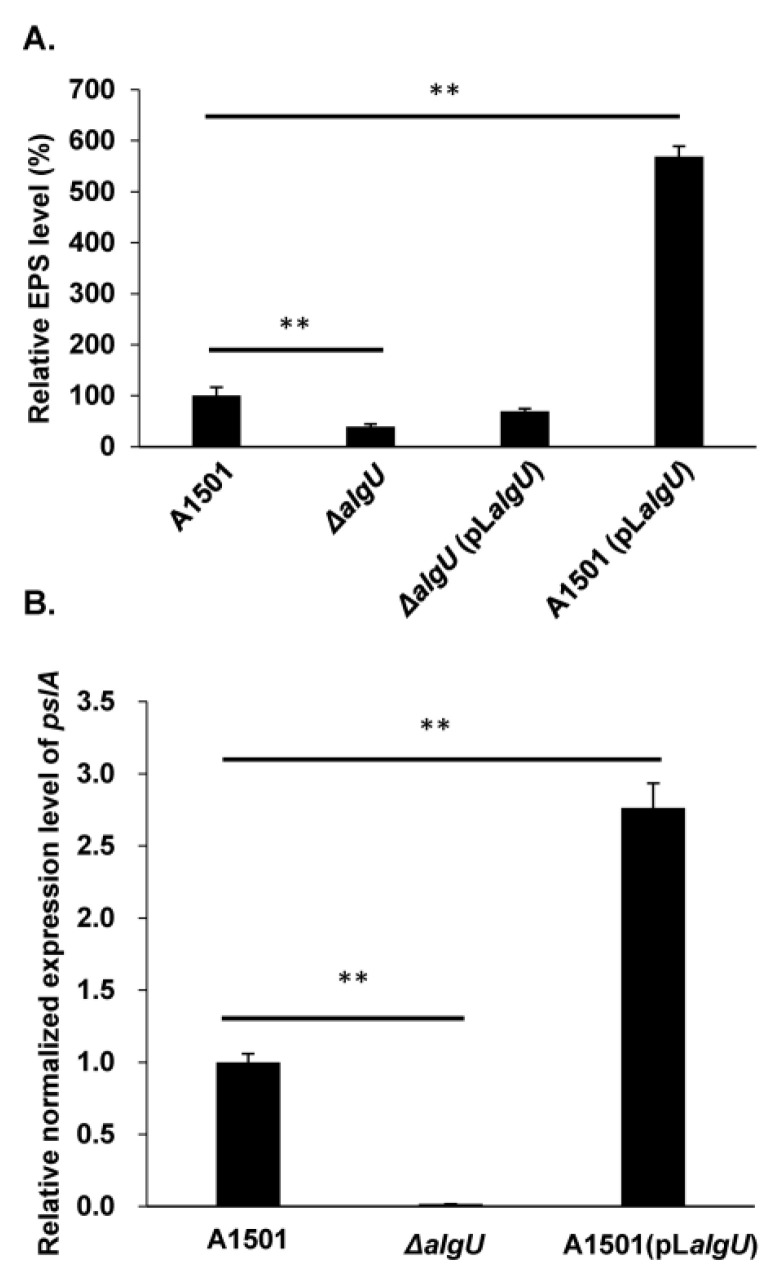
*algU* positively regulates the exopolysaccharide production in *P. stutzeri*. (**A**) Relative exopolysaccharide levels in *P. stutzeri* wild-type A1501, *algU* mutant Δ*algU*, *algU*-complemented strain Δ*algU*(pL*algU*), and *algU* overexpression strain A1501(pL*algU*) determined by the phenol-sulfuric acid method. (**B**) Relative expression levels of *pslA* in the *P. stutzeri* wild-type A1501, *algU* mutant and *algU* overexpression strain were determined by qRT-PCR analysis. Error bars represent the standard deviation (SD) of the three biological replicates from a single experiment. Asterisks indicate statistical significance when compared to wild-type A1501 by one-way ANOVA: ** *p* < 0.01.

Considering that AlgU, as an ECF sigma factor, plays an important role in exopolysaccharide production in *Pseudomonas*, the effect of *algU* mutation and overexpression on exopolysaccharide production was tested in *P. stutzeri* A1501. First, we constructed the *algU* insert mutant strain with the suicide vector pk18mob (Figure S1); then, the low-copy-number plasmid pLAFR3 was used to construct the expression vector pL*algU* carrying the A1501 *algU* gene under the control of the native promoter. When pL*algU* was introduced into the *algU* mutant and A1501, the *algU*-complemented strain and *algU* overexpression strain were obtained, respectively. The mutation of *algU* did not significantly change the growth rate of A1501 in LB medium or K medium with 50 mM lactate and 6 mM NH_4_Cl (Figure S2). In a preliminary experiment, LB medium and minimal K medium were compared for biofilm formation. It was found that A1501 preferentially forms biofilms in minimal K medium supplemented with 50 mM lactate and 6 mM NH_4_Cl (Figure S3), suggesting A1501 produces more exopolysaccharides under this condition. As shown in Figure 1A, the inactivation of *algU* led to a 61% decrease in exopolysaccharide production. In addition, pL*algU* partly complemented exopolysaccharide production in the *algU* mutant, and the *algU* overexpression strain drastically increased exopolysaccharide production by 5.7-fold compared to that of the wild-type strain A1501. The regulation of exopolysaccharide production in A1501 is still largely unclear. Recently, Shang et al. reported that the Psl-like biosynthesis gene *pslA* mutant displayed decreased exopolysaccharide production [26], and a question was raised: does AlgU regulate exopolysaccharide production by changing the expression of *pslA*? To investigate this hypothesis, the transcriptional expression of *pslA* was examined in the *algU* mutant strain and overexpression strain by qRT-PCR analysis. The expression of *pslA* was downregulated by more than 50-fold in the *algU* mutant and was upregulated by 2.8-fold in the *algU*-overexpressing strain (Figure 1B). Based on these data, *algU* positively regulated exopolysaccharide production in A1501.

### 3.2. Inactivation of algU Reduced Biofilm Formation in A1501

**Figure 2:**
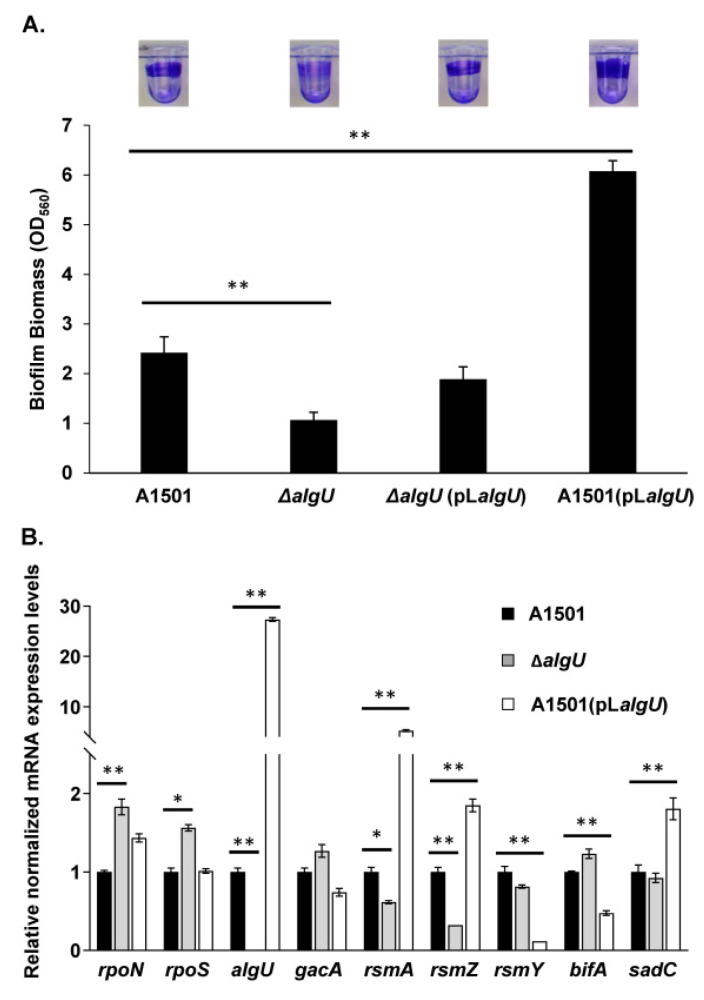
*algU* positively regulates biofilm formation in *P. stutzeri*. (**A**) Biofilm formation of *P. stutzeri* wild-type A1501, *algU* mutant Δ*algU, algU*-complemented strain Δ*algU* (pL*algU*) and *algU* overexpression strain A1501 (pL*algU*) determined by the crystal violet (CV) method. CV staining of the biofilm obtained is shown at the top. (**B**) Relative expression levels of biofilm regulation-related genes in *P. stutzeri* wild-type A1501, *algU* mutant Δ*algU* and *algU* overexpression strain A1501 (pL*algU*) determined by qRT-PCR analysis. Error bars represent the standard deviation (SD) of the three biological replicates. Asterisks indicate statistical significance when compared to wild-type A1501 by one-way ANOVA: * *p* < 0.05; ** *p* < 0.01.

Based on the result above that *algU* positively regulates exopolysaccharide production in A1501, we supposed that *algU* also upregulates biofilm formation in A1501. To test biofilm formation, *P. stutzeri* wild-type A1501, the *algU* mutant, the *algU* complement strain and the *algU* overexpression strain were analyzed by the crystal violet method performed in microtiter plates. A previous study indicated that *P. stutzeri* A1501 tends to form biofilms under nitrogen-deficient conditions [26], hence the lactate containing K medium without any nitrogen was used to perform the biofilm formation test. Inactivation of *algU* reduced biofilm formation by 56%, pL*algU* partly complemented biofilm formation in the *algU* mutant, and biofilm formation increased 6.1-fold in the *algU*-overexpressing strain compared to that in the wild-type strain A1501 (Figure 2A). In addition to the regulation of biofilm formation by affecting exopolysaccharides, AlgU may affect biofilm formation by changing the expression of the Rpo-Gac-Rsm regulatory network. Indeed, the qRT-PCR results in Figure 2B show that mutation of AlgU significantly decreased the expression of *rsmA* and *rsmZ* but mildly increased the expression of *rpoN* and *rpoS*. Notably, *algU* overexpression decreased the expression of *bifA*, a c-di-GMP phosphodiesterase, but increased that of *sadC*, a diguanylate cyclase. The expression of *algU* in A1501, the *algU* mutant and the *algU* overexpression strain was also determined. The expression of *algU* in the *algU* overexpressing strain was near 27-fold of that in the WT strain. This result implies that *algU* may positively upregulate the c-di-GMP level, and it is worthwhile to confirm the relationship between *algU* and the c-di-GMP level in A1501 in future studies.

### 3.3. Inactivation of algU Reduced Biofilm-Based Nitrogenase Activity

**Figure 3:**
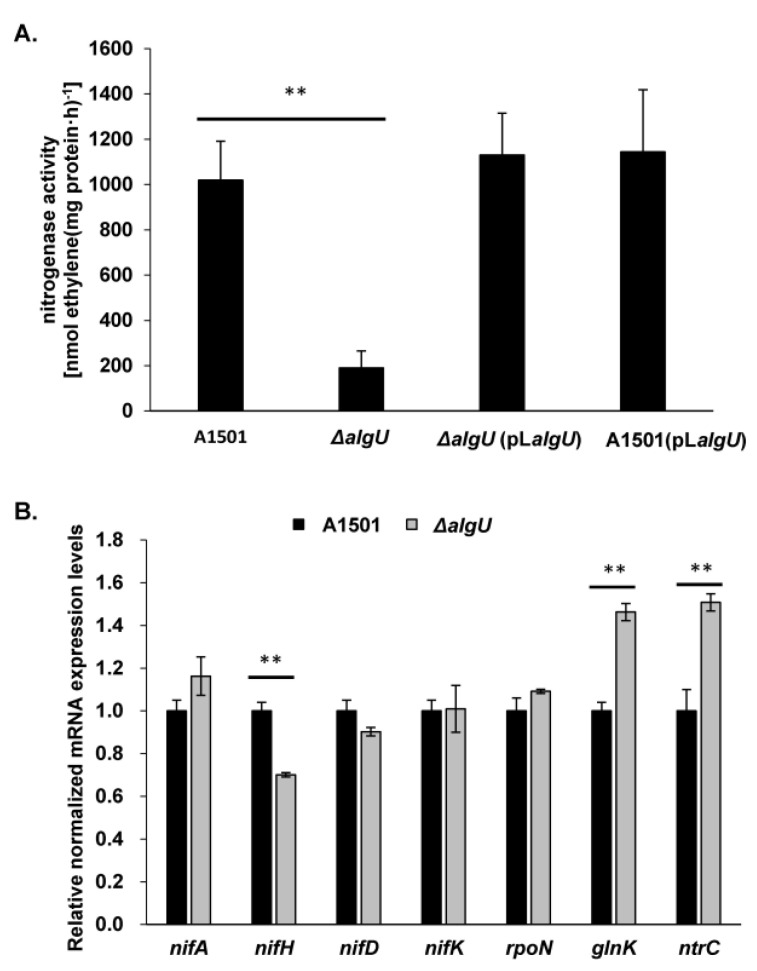
The effect of AlgU on the biofilm-based nitrogenase activity of *P. stutzeri*. (**A**) Biofilm-based nitrogenase activity in wild-type A1501, *algU* mutant Δ*algU*, *algU*-complemented strain Δ*algU* (pL*algU*) and the *algU* overexpression strain A1501 (pL*algU*). (**B**) Relative expression levels of selected nitrogen fixation genes in *P. stutzeri* wild-type A1501 and *algU* mutant Δ*algU* were determined by qRT-PCR analysis. Error bars represent the standard deviation (SD) of the three biological replicates. Asterisks indicate statistical significance when compared to wild-type A1501 by one-way ANOVA: ** *p* < 0.01.

Reports have shown that *algU* plays an important role in survival under various stress conditions. Nitrogen starvation is a common stress for environmental bacteria. The nitrogen fixation ability is advantageous for A1501 in the plant rhizosphere [18]. In addition, the nitrogenase activity is recently reported to be linked with biofilm formation in A1501, it is worthwhile to test whether *algU* affects the nitrogenase activity of A1501 under the nitrogen-fixing biofilm formation condition. The biofilm-based nitrogenase activity test indicated that the mutation of *algU* reduced the biofilm-based nitrogenase activity by 81.4%, and pL*algU* complemented the *algU* mutation (Figure 3A). In addition, the qRT-PCR results indicated that the expression of *nif* genes such as *nifA, nifD and nifK* did not change very much in the *algU* mutant (Figure 3B). Although the expression of the nitrogenase activity regulator *glnK* and *ntrC* were slightly increased (less than 2 times) in the *algU* mutant, the major regulator *rpoN* did not have changed expression in the *algU* mutant. No AlgU-binding motif was found in the upstream region of the *nifLA* and *nifHDK* operons. Therefore, it is unlikely that AlgU affects nitrogenase activity by directly activating the expression of *nif* genes.

### 3.4. AlgU Directly Activated the Transcription of pslA

**Figure 4:**
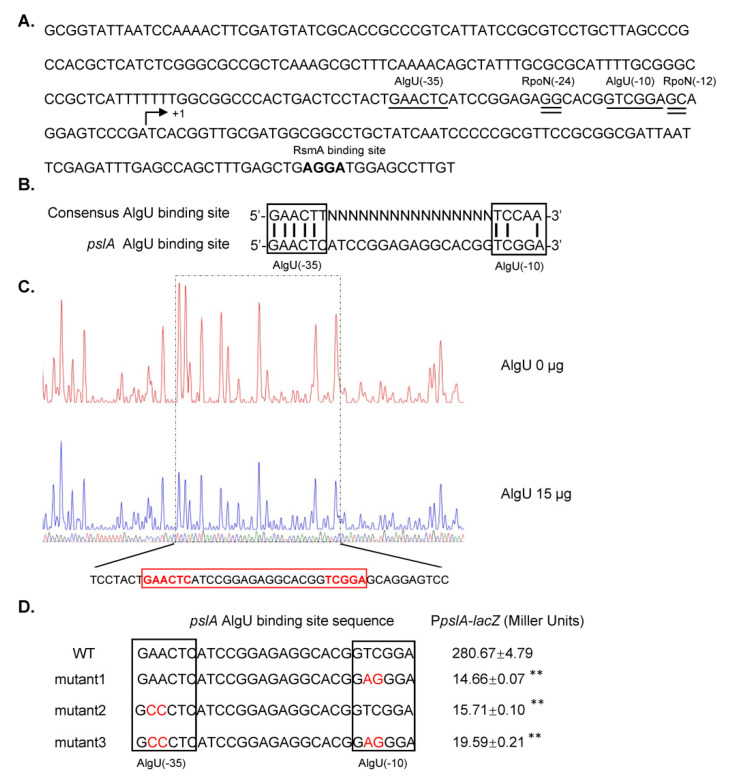
AlgU directly activates the transcriptional expression of *pslA*. (**A**) Promoter sequence analysis of *pslA*. +1 indicates the transcriptional start site of *pslA*. The predicted RpoN-binding site was underlined with a double line, 12–25 bases upstream of the +1 site. The predicted AlgU-binding site was underlined with a single line, 14–40 bases upstream of the +1 site. In addition, the predicted RsmA-binding site is shown in bold font. (**B**) Sequence alignment of the *pslA* AlgU-binding site with the AlgU-binding consensus sequence. (**C**) DNase I footprinting analysis of the *pslA* promoter probe using the purified AlgU protein added at 0 (upper panel) and 15 μg (lower panel). The AlgU-protected region is marked by a dotted box and the DNA sequence is shown at the bottom. The AlgU-binding site is indicated by a red box. (**D**) The β-Galactosidase activities of *lacZ* fusions to the *pslA* promoter region (1–500 bases upstream of the +1 site) with the wild-type AlgU-binding site or mutated AlgU-binding sites. The mutated positions are shown in red. Data are in Miller units and are the means of at least three biological replicates ± standard deviations. Asterisks indicate statistical significance when compared to the WT AlgU-binding site by one-way ANOVA test: ** *p* < 0.01.

As shown in Figure 1B, the mutation of *algU* abolished the transcriptional expression of *pslA*. To test whether *algU* directly regulated the transcription of *pslA*, we inspected the nucleotide sequence upstream of the initiation codon ATG. Interestingly, a 27 bp DNA sequence (GAACTCATCCGGAGAGGCACGGTCGGA) that highly matched the AlgU-binding motif (GAACTT-N16/17-TCCAA) was identified in the promoter region of *pslA* (Figure 4A). This putative AlgU-binding site showed evidence of conserved −10 and −35 boxes separated by a 16 bp spacer region. The location of this putative AlgU-binding site is suitable based on the transcription site previously determined by Shang et al. [26]. In addition, this putative binding sequence overlapped with the previously confirmed RpoN binding site [26]. The AlgU dependent expression of *pslA* was determined by DNase I footprint assays. As shown in Figure 4C, AlgU protects a 44-bp DNA region (TCCTACT**GAACTC**ATCCGGAGAGGCACGG**TCGGA**GCAGGAGTCC) of the *pslA* promoter, which overlapped the putative AlgU-binding site located at positions ranging from −10 to −35 from the transcription start site. In addition, four 500-bp DNA fragments containing the putative wild-type (WT) or mutant (individual nucleotides in −10 or −35 boxes were replaced, as shown in Figure 4D) of AlgU-binding motif and were cloned into the promoter-less *lacZ* reporter vector pGD926 and the β-galactosidase activity were further determined. The results in Figure 4D indicated that the mutations in −10 or −35 boxes drastically reduced the transcriptional activity of the *pslA* promoter. Taken together, these data suggest that AlgU directly activates the transcription of *pslA* in A1501.

### 3.5. Expression of algU was Controlled by RpoN at the Transcriptional Level and RsmA at the Posttranscriptional Level

**Figure 5:**
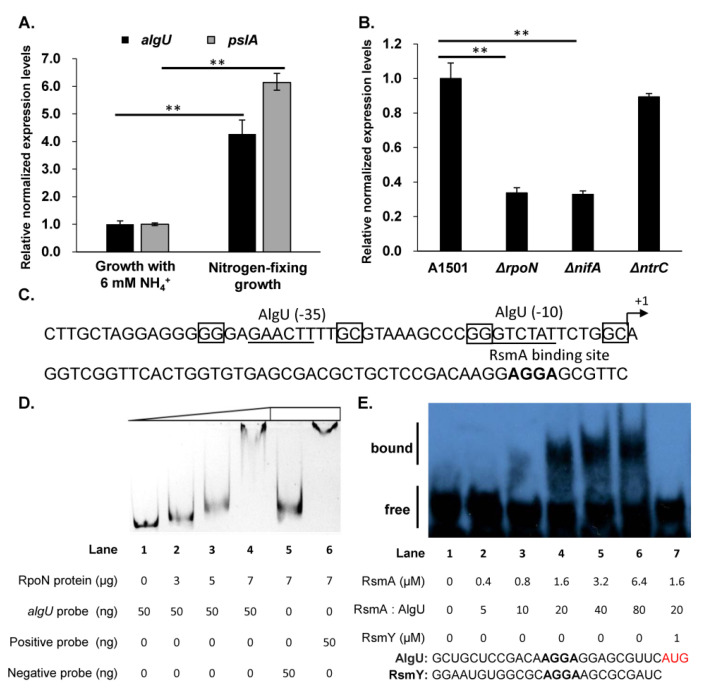
Regulation of the *algU* gene in A1501. (**A**) The relative expression levels of *algU* and *pslA* in *P. stutzeri* wild-type A1501 under two different growth conditions (growth with 6 mM NH_4_^+^: A1501 incubated in minimal K medium containing 50 mM lactate and 6 mM NH_4_^+^ for 4 h under an aerobic atmosphere (O_2_, 21%); nitrogen-fixing growth: A1501 was incubated in minimal K medium containing 50 mM lactate and 0 mM NH_4_^+^ under a microaerobic atmosphere (O_2_, 0.5%) for 4 h). (**B**) Relative expression levels of *algU* in *P. stutzeri* wild-type A1501, *rpoN* mutant Δ*rpoN*, *nifA* mutant Δ*nifA* and *ntrC* mutant Δ*ntrC*. (**C**) Promoter analysis of the *algU* gene. The predicted AlgU-binding site RpoN-binding site and RsmA-binding site were located within the upstream region of the start codon of *algU*. The corresponding −10 and −35 regions of the AlgU-binding site were underlined and the RpoN-binding sites were boxed. +1 indicated the transcriptional start site determined by the 5′ RACE assay. The predicted RsmA-binding site was shown in bold font. (**D**) Gel mobility shift analysis of RpoN and the *algU* promoter. A DNA fragment from *Halomonas venusta* was used as negative control and the *nifA* promoter from A1501 was used as a positive control. (**E**) Gel mobility shift analysis of the RsmA protein with AlgU 5′UTR RNA oligonucleotides. 80 nM AlgU RNA was used to bind the RsmA protein. The positions of free and bound RNA were indicated. The RNA oligonucleotides AlgU and RsmY were synthesized in Genepharma Company. The RsmA binding site AGGA was in bold, and the initiation codon (AUG) for *algU* was indicated in red. Error bars represent the standard deviation (SD) of the three biological replicates. Asterisks indicate statistical significance by one-way ANOVA: ** *p* < 0.01.

Previous experiments have revealed that A1501 increases exopolysaccharide production and biofilm formation under nitrogen-fixing conditions [26]. These phenotypes raise the question of whether the expression of *algU* is activated in response to nitrogen fixation conditions. As shown in Figure 5A, compared to the nitrogen-sufficient growth condition with 6 mM NH_4_Cl, the transcriptional level of *algU* was increased by 4.3-fold under the nitrogen-fixing condition without any nitrogen, in addition, the expression of *pslA* also was upregulated by 6.1-fold. Moreover, qRT-PCR analysis was also performed among the wild-type A1501 and three mutants (Δ*rpoN*, Δ*ntrC* and Δ*nifA*), which contained mutations in genes that mainly control nitrogen fixation. The expression of *algU* significantly decreased by 3-fold in the *rpoN* and *nifA* mutants. The determination of the *algU* transcription initiation site with a 5′RACE assay showed that the transcript started from an adenine residue, which is the 50th nucleotide upstream from the ATG initiation codon. A putative AlgU-binding site (GAGAACTTTTGCGTAAAGCCCGGGTCTATTC) and two putative RpoN-binding sites (GGN_10_GC) were predicted in the promoter region of *the algU* gene; however, no binding site for NifA was found. The locations of the two putative RpoN-binding sites were both atypical (most typical RpoN-binding sites are located from −12 to −24 upstream from the transcription start site). The gel mobility shift analysis was used to detect the interaction between RpoN protein and *algU* promoter DNA, and it was found that the RpoN protein could specifically bind to the *algU* promoter DNA (Figure 5D); besides this, self-regulation of *algU* was also been reported in *P. aeruginosa*. In addition, a putative RsmA-binding site (one GGA motif) was predicted, which overlapped with the ribosome-binding site in the *algU* 5′UTR sequence. The purified RsmA protein was also found to significantly change the mobility of the RNA band, the binding between RsmA protein and AlgU RNA gradually increased as the concentration of RsmA increased (Figure 5E). The RsmA competitor RNA RsmY could compete effectively for RsmA indicating the RsmA binded AlgU specifically (Figure 5E). The same regulation of AlgU by RsmA was also reported in *P. aeruginosa*. To subsequently confirm the posttranscriptional regulation of AlgU by RsmA in A1501, the pGD926 vector was used to construct the AlgU-flag expression vector pG*algU*-flag with 500 bp native promoter. pG*algU*-flag was introduced into A1501, RsmA mutant and GacA mutant, individually. Western blot analysis indicated that the AlgU protein level was increased in the RsmA mutant and decreased in the GacA mutant (Figure S4). The qRT-PCR analysis indicated the transcriptional expression of *algU* was slightly reduced in the RsmA mutant compared to A1501 (Figure S4). The data described above suggest that under nitrogen fixation conditions, RpoN binds the *algU* promoter and activates the expression of *algU*, however, *algU* is posttranscriptionally repressed by RsmA protein.

## 4. Discussion

*P. stutzeri* A1501 is a root-associated nitrogen-fixing bacterium isolated from rice paddies [18]. Compared to the rhizobia, the association between A1501 and host roots is easily affected by various environmental stresses, which leads to a limited nitrogen fixation efficiency in the rhizosphere. Promoting the biofilm formation of A1501 in plant roots will be useful for successful root surface colonization. It is very important to analyze the regulatory mechanism of exopolysaccharide synthesis and biofilm formation for the development of genetically engineered bacteria with more colonization in plant roots and higher nitrogen fixation levels [2]. AlgU, as one of the ECF sigma factors in gram-negative bacteria, is essential for the survival of bacteria under various stress environments [35]. In *Pseudomonas aeruginosa*, AlgU regulates the expression of genes related to alginate biosynthesis enzymes [36], which contributes to biofilm formation and resistance to various external stresses, such as oxidative stress, osmotic stress, and desiccation stress [37]. When grown in the soil or the rhizosphere, *P. stutzeri* A1501 is faced with various environmental stresses; these stresses reduce nitrogen fixation, and therefore, we speculate that AlgU in A1501 could regulate various types of stress responses and optimize nitrogen fixation.

**Figure 6:**
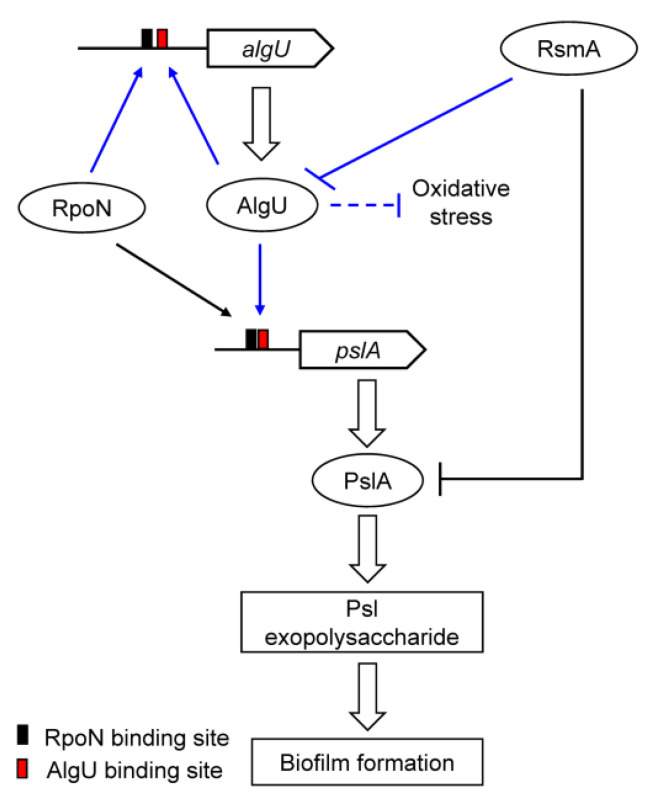
Proposed regulatory model for the *P. stutzeri* A1501 AlgU controlling exopolysaccharide synthesis and nitrogen-fixing biofilm formation. In this model, AlgU directly activates the transcription of *pslA*. PslA plays an essential role in the production of Psl exopolysaccharides and biofilm formation. Additionally, AlgU and PslA are positively regulated by RpoN at the transcriptional level and negatively regulated by RsmA at the posttranscriptional level. Arrows and T-shaped bars indicate positive and negative regulation, respectively. Broken lines indicate regulations for which evidence exists but that need to be studied in further detail. Black and blue lines represent results obtained in previous studies and this study, respectively.

So far, no evidence has shown that AlgU is directly involved in the regulation of *psl* genes. In the present work, it was shown that mutation of *algU* significantly reduced exopolysaccharide production and biofilm formation, together with the biofilm-based nitrogenase activity. A predicted AlgU-binding site was identified in the promoter region of *pslA*. Both the DNase I footprinting assays and β-galactosidase activity analysis indicated that AlgU could directly activate the expression of *pslA*. The regulation of *algU* expression and the role of AlgU in the regulation of nitrogen-fixing biofilm formation in *P. stutzeri* A1501 are summarized in Figure 6. AlgU promotes exopolysaccharide production and biofilm formation by directly activating the transcription of *pslA*. In addition, AlgU and PslA are positively regulated by RpoN at the transcriptional level and negatively regulated by RsmA at the posttranscriptional level.

Although exopolysaccharide composition, construction, and biofilm formation regulation vary greatly among the different *Pseudomonas* species, the basic regulatory network is conserved. The basic network is reported to include three parts: (1) the quorum-sensing pathway, (2) the Gac-Rsm pathway, and (3) the c-di-GMP signalling pathway. Recently, the Rpo-Gac-Rsm network was reported to regulate nitrogen-fixing biofilm formation in A1501, and RpoN plays a key role [26]. Crosstalk between AlgU and the Rpo-Gac-Rsm pathway has been reported in *P. aeruginosa*. First, AlgU acts as a regulator to directly activate the transcriptional expression of *rsmA* [34]. Second, RsmA represses the expression of AlgU by binding the RsmA-binding site overlapping the SD region in AlgU mRNA [38]. Third, *algD* expression is regulated by AlgU and RpoN [39]. RpoN represses AlgU-dependent *algD* transcription by directly binding the *algU* promoter, and the binding sites for AlgU and RpoN overlap upstream of the *algD* transcription start site. Notably, RpoN is required as a positive factor for *algD* expression in the *P. aeruginosa* muc23 strain, in which AlgU affects the transcription of *algD* [38]. Interestingly, the RpoN- and AlgU-binding sites were also found in the upstream region of the *pslA* transcriptional start site, and these two sites overlapped (Figure 4A). RpoN was previously reported to activate the transcription of *pslA* in A1501 [26]. In this study, AlgU was also proven to activate the transcription of *pslA*. Under some conditions or in some specific strains, the possible regulatory mechanism of AlgU and RpoN competing with each other to activate or repress the expression of *pslA* could not be excluded.

The complex regulation mode between AlgU and RsmA in *P. stutzeri* A1501 is similar to that in *P. aeruginosa*: AlgU upregulated the expression of *rsmA* (Figure 2B), and RsmA repressed the expression of AlgU (Figure S4). Although AlgU positively regulates the expression of *rsmA*, it could not regulate Psl production or biofilm formation through mainly RsmA because RsmA was previously reported to repress *pslA* expression and biofilm formation in A1501 [26]. Schulz et al. reported the sigma network had a modular structure with limited and function-specific crosstalk in *P. aeruginosa* and the quantitative analysis of sigma factor regulons indicated RpoN and AlgU co-regulate the expression of 49 genes [40]. In addition, the expression of *algU* reduces by more than 2 times in *rpoN* mutant, however, no RpoN-binding site is found in the promoter region of *algU* [40]. In this study, we found the expression of *algU* was partly controlled by RpoN, interestingly, two RpoN-binding sites were predicted. The locations of predicted RpoN-binding sites are not the −12 and −24 positions, which implies that these RpoN-binding sites are untypical or fake. The purified RpoN specifically binds the *algU* promoter DNA indicated in Figure 5D, which gives us more confidence that RpoN directly activates the transcription of *algU*. It is not clear which pathway AlgU is involved in that mainly affects biofilm-based nitrogenase activity. Because the biofilm formation could enable A1501 to fix nitrogen under the aerobiotic condition and the *algU* mutation reduced the biofilm formation, it is reasonable to speculate the AlgU could protect the nitrogenase from oxygen or active oxygen species. The significant change in the expression of several oxidative stress response genes in the AlgU mutant implies the possibility that the AlgU mutation increases the amount of active oxygen that would repress nitrogenase activity (Figure S5). To our knowledge, among the *Pseudomonas* species, *pslA* is the new exopolysaccharide synthesis gene that has been determined to be directly activated by AlgU. The *pslA* gene is conserved and widespread in *P. aeruginosa*, *P. stutzeri*, *P. protegens*, *P. fluorescens* and so on. The expression of *pslA* is reduced in the *P. aeruginosa* AlgU mutant; however, no obvious match to the AlgU consensus promoter element is found in the upstream region of *pslA* [11], and therefore, whether the direct regulation between AlgU and PslA is is conserved among *Pseudomonas* species is worth further study.

## Data Availability

Not applicable.

## Supplementary Materials

The following supporting information can be downloaded at: www.mdpi.com/article/10.3390/genes13050867/s1, Table S1: Strains and plasmids used in this study; Table S2: Primers used in this study; Figure S1: Validation of the *algU* mutant; Figure S2: The effect of *algU* mutation on the growth of *P. stutzeri*; Figure S3: Biofilm formation ability of *P. stutzeri* A1501 in LB and K medium; Figure S4: The expression level of *algU* is regulated by the Gac/Rsm system; Figure S5: The relative normalized expression levels of several oxidative stress response genes in the *algU* mutant.
