# Supplementary material for: The Sigma Factor AlgU Regulates Exopolysaccharide Production and Nitrogen-Fixing Biofilm Formation by Directly Activating the Transcription of pslA in Pseudomonas stutzeri A1501
Source: Genes (Basel). 2022 May 12;13(5):867. doi: 10.3390/genes13050867 (PMC9141998; doi:10.3390/genes13050867)
Supplement: Supplementary file 1 [file genes-13-00867-s001.zip › genes-1614025-supplementary.pdf]

The sigma factor AlgU regulates exopolysaccharide production and nitrogen-fixing biofilm formation by directly activating the transcription of *pslA* in *Pseudomonas stutzeri* A1501

Yahui Shao [1], Changyan Yin [1], Fanyang Lv [1], Shanshan Jiang [1], Shaoyu Wu [1], Yueyue Han [1], Wei Xue [1], Yiyuan Ma [1], Juan Zheng [1], Yuhua Zhan [1], Xiubin Ke [1], Wei Lu [1], Min Lin [1], Liguo Shang [2,*] and Yongliang Yan [1,*]

[1]Biotechnology Research Institute, Chinese Academy of Agricultural Sciences, Beijing 100081, China

[2]School of Basic Medicine, GuangXi University of Chinese Medicine, Nanning, 530200, China

*Correspondence: shanglg@gxtcmu.edu.cn (L.S); yanyongliang@caas.cn (Y.Y.)

Supplementary Materials:
Table S1: Strains and plasmids used in this study;
Table S2: Primers used in this study;
Figure S1: Validation of the *algU* mutant;
Figure S2: The effect of *algU* mutation on the growth of *P. stutzeri*;
Figure S3: Biofilm formation ability of *P. stutzeri* A1501 in LB and K medium;
Figure S4: The expression level of *algU* is regulated by the Gac/Rsm system;
Figure S5: The relative normalized expression levels of several oxidative stress response genes in the *algU* mutant.

Table S1. Strains and plasmids used in this study.

Strains or plasmids	Relevant characteristics	Source or reference
Strains		
Pseudomonas stutzeri		
A1501	WT, Chinese culture collection: CGMCC 0351	Lab collection
Δ*algU*	*algU* insertion mutant, Km^R	This study
Δ*algU*(pL*algU*)	*algU* insertion mutant containing pL*algU*, Km^R and Tc^R	This study
A1501(pL*algU*)	A1501 containing pL*algU*, Tc^R	This study
Δ*rpoN*	*rpoN* deletion mutant, Cm^R	Desnoues N, et al. 2003
Δ*nifA*	*nifA* deletion mutant, Cm^R	Desnoues N, et al. 2003
Δ*ntrC*	*ntrC* deletion mutant, Cm^R	Desnoues N, et al. 2003
Δ*rsmA*	*rsmA* insertion mutant, Km^R	Shang L, et al.2021
Δ*gacA*	*gacA* deletion mutant, Cm^R	Shang L, et al.2021
Escherichia coli		
DH5α	Competent cell for cloning	Vazyme Biotech Company

Plasmids		
pK18mob	Mobilizable plasmid containing an *E. coli* origin of replication, Km^R.	Schäfer et al. 1994
pK18mob-*algU*	pK18mob derivative carrying the A1501 *algU* insertion fragment, Km^R	This study
pRK2013	Helper plasmid for conjugation into *P. stutzeri* , Km^R	Figurski et al. 1979
pLAFR3	For complement strain construction, Tra-, mob+, cos, RK2 replicon, Tc^R	Lab collection
pL*algU*	pLAFR3 derivative carrying the *algU* gene under the control of its endogenous promoter, Tc^R	This study
pGD926	For β-galactoside activity test of the promoter region. Tra-, mob+, cos, RK2 replicon, Tc^R	Lab collection
pG*algU*-flag	pGD926 derivative carrying the *algU* gene with flag tag under the control of its endogenous promoter, Tc^R	This study
pG*pslA*-WT-*lacZ*	pGD926 derivative carrying the wild type *pslA* promoter region	This study
pG*pslA*-mutant1-*lacZ*	pGD926 derivative carrying the *pslA* promoter region with mutation	This study
pG*pslA*-mutant2-*lacZ*	pGD926 derivative carrying the *pslA* promoter region with mutation	This study
pG*pslA*-mutant3-*lacZ*	pGD926 derivative carrying the *pslA* promoter region with mutation	This study

Table S2. Primers used in this study.

Primer's Name	Sequence (5′-3′)	Purpose
nifL-rnfA F	TCAGCTGGCCGAGAAGGG	Assessment of genomic
nifL-rnfA R	TCAGATCAGGCCGGCGAAGC	DNA contamination
RT-16s-F	CCTACGGGAGGCAGCAG	qRT-PCR
RT-16s-R	ATTACCGCGGCTGCTGG	
RT-*pslA*-F	CTCACCCGAGTTTCGTCGAT	
RT-*pslA*-R	ATGATGACGCTGAGTACCGC	
RT-*algU*-F	TGCGTACGGCTCTAACACTG	
RT-*algU*-R	TATCTATGGCTTCACGCGCC	
RT-*rpoN*-F	CTTCTTCTCCAGCCACGTCAG	
RT-*rpoN*-R	CCAGTAAACCAGCGATCTTGC	
RT-*rpoS*-F	GCATGATCGAAAGCAACCTG	
RT-*rpoS*-R	CAGATAGACATTCAGCTCCTTCAC	
RT-*gacA*-F	GAAGAGATGGTGCAGGCCA	
RT-*gacA*-R	TTTCCCGTTCGGAAAGCAGA	
RT-*rsmA*-F	GTGGGTGACGATGTGACTGT	
RT-*rsmA*-R	GTGGCTTGGTTCCTGATCCT	
RT-*rsmZ*-F	CTCAGGACGAGGGTCAGGA	
RT-*rsmZ*-R	TCCCTGTTCCCTGTATCCCTT	
RT-*rsmY*-F	GCGATCAAACAACACGGACC	
RT-*rsmY*-R	GGGCTCTGCAGACTGAATCC	
RT-*bifA*-F	GAAGGCGTCGAAACTCCTGA	
RT-*bifA*-R	GGCAGGGGCTTGCTGTAATA	
RT-*sadC*-F	TGGCGAGTGGTTTCTACGAG	
RT-*sadC*-R	ATAGAGCACGAGCAGTGAGC	
RT-*nifA*-F	CGCGAAGACCTCTACTACCG	
RT-*nifA*-R	CAGCTTGAGTTTGCGACCCT	
RT-*nifH*-F	GAGATGATGGCGATGTATGC	
RT-*nifH*-R	GGTCGGTGTTGCGGCTGTTG	
RT-*nifD*-F	ACATGATCCACATTTCCCACG	
RT-*nifD*-R	GAACAGCGTCTCGATCTCGTC	
RT-*nifK*-F	TCGAGACCTACCTGGGCAACT	
RT-*nifK*-R	GGGGTATCGAGCACTTCTTCC	
RT-*ntrC*-F	GATCAATGGCGAATCGGGTAC	
RT-*ntrC*-R	CAGCTCGGATTCCATCAGGTC	
RT-*glnK*-F	AGTCACTGCCATCATCAAGCC	
RT-*glnK*-R	GCCACGTCGATCTTCACCTTT	
RT-*katA*-F	ATGGACCAATCTGAAGAGCC	
RT-*katA*-R	CGTGCATGAACCGATAACC	
RT-*katB*-F	CTTCTTGCTGAACGAGCGATAC	
RT-*katB*-R	TTCTCCTACGCCGATACCCA	
RT-*katE*-F	GCTGGACCCGACCAAAAT	
RT-*katE*-R	CGGACGGTTGATCGGAAT	

RT-*katG*-F	TTCCGCAACTACTACCACGAG	
RT-*katG*-R	TGTCCAGCAGGTTGACGAAG	
RT-*oxyR*-F	TCATACAGCGGCTTGGTCA	
RT-*oxyR*-R	TACATGTTCCCGCACCTGAT	
RT-*ahpC*-F	GTCTTCTCGCCCTCTTTCCA	
RT-*ahpC*-R	AGATCGCTCGTGACGTGTCC	
RT-*nfiR*-F	CTGACCCTCAACGTGG	
RT-*nfiR*-R	CTTTGTCCGTGTCCCG	
RT-*nfiS*-F	ACTGCTGATCCATCTGCTGAG	
RT-*nfiS*-R	CTGCATCAGCGGGCAATG	
M-*algU*-F	GGAAACAGCTATGACATGATTACGATGCTGACTCAGGAGCAGGAC	*algU* mutant construction
M-*algU*-R	CATGCCTGCAGGTCGACTCTAGAGCCGCGAAAATTGGCGAGTGCGCG	
pK18mob-test-F	GCCGATTCATTAATGCAGCTGGCAC	Validation of *algU* mutant
algU-test-R	AGAGCCGTACGCAAATCTTCTGG	
C- *algU*-F	AAACAGCTATGACCATGATTACGAACTCCGCCCTGAGCCCAATAGG	*algU* complement plasmid construction
C- *algU*-R	CCAAGCTTGGCTGCAGGTCGACGTCAGGATTCATGCAACAAGGGTTC	
pG*pslA*-WT-F	ATCAGGCGATTGTCGAAGCTTGGCTCCCAGGTGACACGAA	Primers for the construction of the pG*pslA* vector
pG*pslA*-WT-R	CGTTGTAAAACGACGGGATCTCCAACAGACTGTACGCGCA	
pGp*slA*-mutant1-F1	ATCAGGCGATTGTCGAAGCTTGGCTCCCAGGTGACACGAA	Primers for the construction of the pG*pslA*-mutant1 vector
pG*pslA*-mutant1-R1	AACCGTGATCGGGACTCCTGCTCCCTCCGTGCCTCTC	
pGp*slA*-mutant1-F2	CAGGAGTCCCGATCACGGTTG	
pG*pslA*-mutant1-R2	CGTTGTAAAACGACGGGATCTCCAACAGACTGTACGCGCA	
pGp*slA*-mutant2-F1	ATCAGGCGATTGTCGAAGCTTGGCTCCCAGGTGACACGAA	Primers for the construction of the pG*pslA*-mutant2 vector
pG*pslA*-mutant2-R1	CTCTCCGGATGAGGGCAGTAGGAGTCAG	
pGp*slA*-mutant2-F2	TACTGCCCTCATCCGGAGAGGCACGGTCGGAG	
pG*pslA*-mutant2-R2	CGTTGTAAAACGACGGGATCTCCAACAGACTGTACGCGCA	
pGp*slA*-mutant3-F1	ATCAGGCGATTGTCGAAGCTTGGCTCCCAGGTGACACGAA	Primers for the construction of the pG*pslA*-mutant3 vector
pG*pslA*-mutant3-R	CTCTCCGGATGAGGGCAGTAGGAGTC	

1	AG	
pG*pslA*-mutant3-F<br>2	TACTGCCCTCATCCGGAGAGGCACGG<br>AGGGAGC	
pG*pslA*-mutant3-R<br>2	CGTTGTAAAACGACGGGATCTCCAAC<br>AGACTGTACGCGCA	
AlgU-flag-F<br><br>AlgU-flag-R	CAGCTATGACCATGATTACGAACTCC<br>GCCCTGAGCCCAATAG<br>GCTTGGCTGCAGGTCGACGTTACTAT<br>TTATCGTCGTCATCTTTGTAGTCGATA<br>TCATGATCTTTATAATCACCGTCATGG<br>TCTTTGTAGTCGGATTCATGCAACAA<br>GGGTTGC	Primers for the construction of the *algU*-flag expression vector (FLAG tag sequence is underlined)
algU-GSP1<br><br>*algU*-GSP2	GATTACGCCAAGCTTCTCACATCACT<br>GTCCGGCGGACGCCTT<br>GATTACGCCAAGCTTACGATCAACCC<br>AAGGATCTTGTGCTG	For 5′RACE
pJET1.2-F<br>pJET1.2-R	CGACTCACTATAGGGAGAGCGGC<br>AAGAACATCGATTTTCCATGGCAG	Universal primer
pJET1.2F2<br>pJET1.2R2	AACTTCGATGTATCGCACC<br>CAAGGAAGTAATCGACGAAACT	For DNase I footprint assay of AlgU protein and *pslA* promoter probe
EMSA-*algU*-F<br>EMSA-*algU*-R	AGTTTGCTATGGACACCAGG<br>ACGCTGCACTCGTTCAAC	For the gel mobility-shift assay for RpoN protein and *algU* promoter probe
EMSA-*nifA*-F<br>EMSA-*nifA*-R	CGACTCACTATAGGGAGAGCGGC<br>AAGAACATCGATTTTCCATGGCAG	For the gel mobility-shift assay for RpoN protein and *nifA* promoter probe
EMSA-*nc*-F<br>EMSA-*nc*-R	TGTAAAACGACGGCCAGT<br>CAGGAAACAGCTATGACC	For the gel mobility-shift assay for RpoN protein and negative control probe

Figure S1: Validation of the *algU* mutant

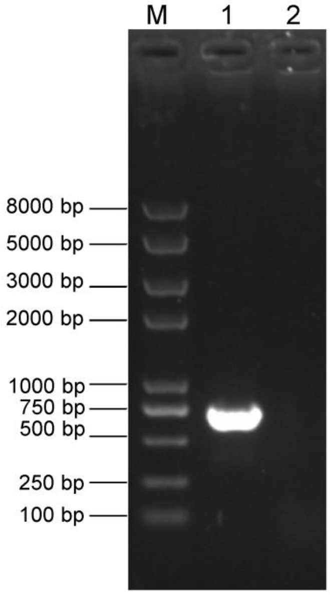

Figure S1. Validation of the *algU* mutant. Lane 1: the *algU* mutant; Lane 2: the wild type A1501; M: Trans2K Plus II DNA Marker. PCR amplification from *algU* mutant gave a strong single band of 630 bp as expected.

Figure S2: The effect of *algU* mutation on the growth of *P. stutzeri*

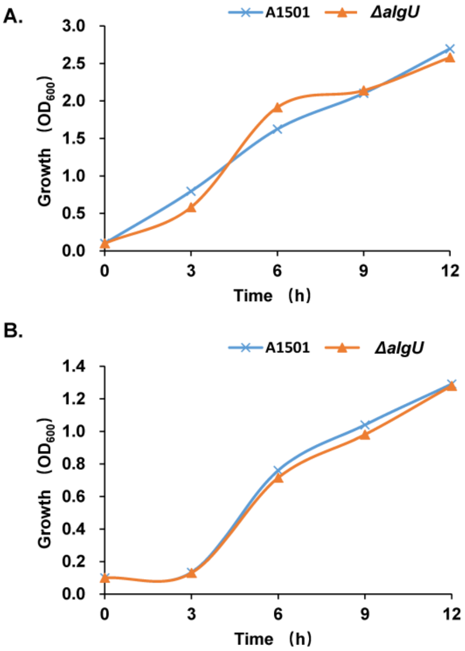

Figure S2. The effect of *algU* mutation on the growth of *P. stutzri*. (A) Growth curve of *P. stutzeri* A1501 and the *algU* mutant in rich LB medium. (B) Growth curve of *P. stutzeri* A1501 and the *algU* mutant in minimal K medium containing 50 mM lactate and 6 mM NH_4Cl.

Figure S3: Biofilm formation ability of *P. stutzeri* A1501 in LB and K medium

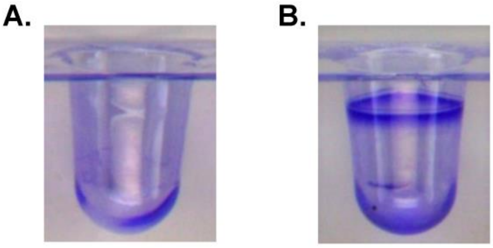

Figure S3. Biofilm formation ability of *P. stutzeri* A1501 in LB medium (A) and minimal medium K containing 50 mM lactate and 6 mM NH_4Cl (B).

Figure S4: The expression level of *algU* is regulated by the Gac/Rsm system

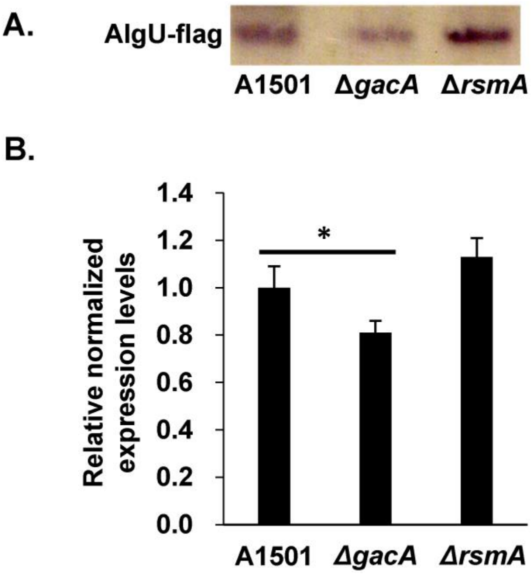

Figure S4. The expression level of *algU* is regulated by the Gac/Rsm system. (A)Western blot analysis of the AlgU-flag protein in *P. stutzeri* A1501, the *gacA* mutant and the *rsmA* mutant. (B) Relative expression levels of *algU* in *P. stutzeri* A1501, the *gacA* mutant and the *rsmA* mutant by qRT-PCR analysis. Error bars represent the standard deviation (SD) of the three biological replicates. Asterisks indicate statistical significance when compared to wild-type A1501 by one-way ANOVA: *$p < 0.05$.

Figure S5: The relative normalized expression levels of several oxidative stress response genes in the *algU* mutant

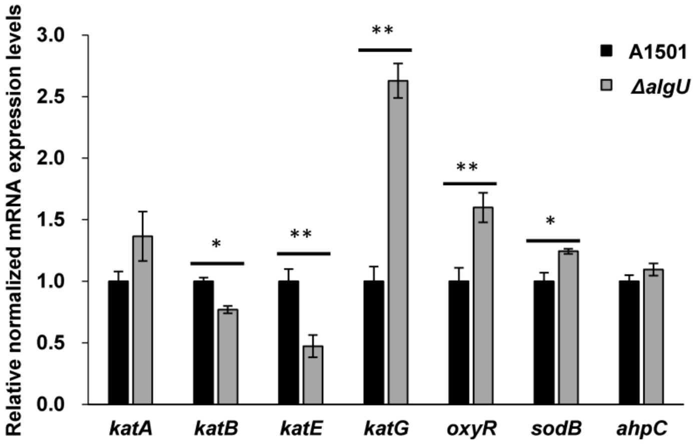

Figure S5. The relative normalized expression levels of several oxidative stress response genes in the *algU* mutant. Asterisks indicate statistical significance when compared to wild-type A1501 by one-way ANOVA: *$p < 0.05$; **$p < 0.01$.

References

Desnoues N, Lin M, Guo X, Ma L, Carreno-Lopez R, Elmerich C. Nitrogen fixation genetics and regulation in a *Pseudomonas stutzeri* strain associated with rice. Microbiology (Reading). 2003, 149(Pt 8):2251-2262.

Figurski DH, Helinski DR. Replication of an origin-containing derivative of plasmid RK2 dependent on a plasmid function provided in trans. 1979, Proc Natl Acad Sci USA. 76(4): 1648–1652.

Schäfer A, Tauch A, Jäger W, Kalinowski J, Thierbach G, Pühler A. Small mobilizable multi-purpose cloning vectors derived from the *Escherichia coli* plasmids pK18 and pK19: selection of defined deletions in the chromosome of *Corynebacterium glutamicum*. 1994, Gene. 145(1): 69–73.

Shang L, Yan Y, Zhan Y, Ke X, Shao Y, Liu Y, Yang H, Wang S, Dai S, Lu J, Yan N, Yang Z, Lu W, Liu Z, Chen S, Elmerich C, Lin M. A regulatory network involving Rpo, Gac and Rsm for nitrogen-fixing biofilm formation by *Pseudomonas stutzeri*. NPJ Biofilms Microbiomes. 2021, 7(1):54.
